# Predicting the potential risk area of illegal vaccine trade in China

**DOI:** 10.1038/s41598-017-03512-3

**Published:** 2017-06-20

**Authors:** Yilan Liao, Yanhui Lei, Zhoupeng Ren, Huiyan Chen, Dongyue Li

**Affiliations:** 1 The State Key Laboratory of Resources and Environmental Information System, Institute of Geographic Sciences and Natural Resources Research, Chinese Academy of Sciences, Beijing, 100101 China; 2 The Key Laboratory of Surveillance and Early-Warning on Infectious Disease, Chinese Center for Disease Control and Prevention, Beijing, 102206 China; 3 Geomatics College, Shandong University of Science and Technology, Qingdao, 266590 China; 4 The School of Earth Science and Resources, Chang’an University, Xi’an, 710054 China

## Abstract

Since the disclosure of the “Illegal vaccine operation series case in Jinan, Shandong” in March, 2016, this issue has attracted a great deal of attention and has led to public concerns about the safety and efficacy of the vaccines involved in this case. The main purpose of this paper is to scientifically and scrupulously predict the possible geographic distribution of illegal vaccines in China, and provide a foundation to guide future governmental policies and actions. A species distribution model was used because of the advantages of using presence/pseudo-absence or presence-only data, and it performs well with incomplete species distribution data. A series of socioeconomic variables were used to simulate habitat suitability distribution. Maxent (Maximum Entropy Model) and GARP (Genetic Algorithm for Rule set Production) were used to predict the risks of illegal vaccines in China, and define the spatial distribution and significant factors of the area at risk from illegal vaccines. Jackknife tests were used to evaluate the relative importance of socioeconomic variables. It was found that: (1) Shandong, Hebei, Henan, Jiangsu and Anhui are the main high-risk areas impacted by the vaccines involved in Jinan case. (2) Population density and industrial structure are the main socioeconomic factors affecting areas which may be at risk from illegal vaccines.

## Introduction

In April 2015, the Food and Drug Environmental Crime Investigation Detachment of the Jinan Public Security Bureau of Shandong, together with the Auditing Detachment of the Food and Drug Administration, uncovered illegal vaccine operation case committed by Pang X and Sun X (a mother and daughter)^1^. 45 pharmaceutical operations were involved in this case, and the illegal vaccines flowed into 59 vaccination units^1^. Illegal vaccines are self-paid vaccines that the citizen receiving the vaccination pays for them, in contrast to those included in the Expanded Program on Immunization. The list of sealed-up vaccine varieties included 12 kinds of illegal vaccines, and the number of vaccines involved may have been as high as 23,000^2^. The affected vaccines flowed to partial vaccination units in nearly 80 counties and cities in 24 provinces, including Shandong, Beijing, Anhui and others^3^. According to the official investigation, the vaccines involved were manufactured by normal manufacturers and were qualified vaccines if used within the expiration date, but they were not kept in cold storage throughout transportation. Although the committee experts comprehensively analysed the thermal stability of the vaccines, the verification of the checked and detained vaccines, the monitoring of adverse vaccination reactions, and the current data on infectious diseases and field investigations, it was concluded that the vaccines did not create any risks beyond normal adverse reactions and that the vaccines involved were still effective^2^. However, a comprehensive and exhaustive investigation, which included tracing the sales and use of these illegal vaccines, was challenging, making it difficult for the government to react to this potential public health threat. Because the vaccines involved were transported to other provinces (regions and cities) and the exact flow direction was uncertain except at a few specific inflow points, this paper evaluates the areas which were at risk of being impacted by the use of these vaccines. If such an event occurs in the future, it will be important to identify the affected geographic areas so that the government could take appropriate action.

In ecology, species distribution models (SDMS) are usually adopted to examine the adaptability distribution of a species in a certain region. The distribution area is estimated using mathematical models based on the recorded data of the species and spatial characteristics or environmental features of potential regions^4^. SDMS are used to predict the range of plant diseases and insects, model species distribution or communities or ecological systems, assess the impact of climate, land utilization and other environmental changes on species distribution^5, 6^, evaluate the risk of species invasion and proliferation^7, 8^, identify highly suitable regions for endangered species in unexplored areas^9^, assist in establishing natural reserve areas^10^, identify species in need of protection in specific areas and reintroduce species to specific areas^11, 12^. Typical SDMS include Maxent^13^, GARP^14, 15^, BIOCLIM^16^, DOMAIN^14, 17^, GAM^14, 18^, GLM^19^ and BIOMAPPER^20^. These models use presence/pseudo-absence or presence-only data to successfully predict the potential distribution of specific species of plants and animals in many regions. However, SDMS models differ in concepts, underlying assumptions, advantages and limitations^21^. Maxent and GARP outperform other classical modeling approaches, such as DOMAIN, BIOCLIM, and Logistic Regression, in their ability to make accurate predictions in simulations and evaluations using presence-only data^21–23^. Maxent and GARP were used to simulate the habitat suitability distribution in this study. Elith *et al*.^24^ used 16 methods to model the potential distributions of 226 species from six areas around the globe. The results showed that the predictive ability of Maxent model was always stable and reliable, and it had a better performance than several SDMS (such as GLM, GAM, DOMAIN, and BIOCLIM)^12^. These models were chosen because both can use presence-only data and perform well with incomplete species distribution data and a series of environment variables. Even more importantly, Maxent and GARP can characterize the geographical distributions of species using small sample sizes. Maxent can predict potential distributions of species with sample sizes as low as five, and GARP produces accurate results at even lower sample sizes (more than10)^25^.

In this paper, we can only get the illegal vaccine inflow point data with presence-only data and a few socioeconomic variable data. Therefore, in this paper, GIS technology and the Maxent and GARP models are used, with the illegal vaccine inflow point data and the corresponding socioeconomic variable data, to predict the geographical distribution of illegal vaccine risk areas. An illegal vaccine inflow region risk map was also created. The potential range of illegal vaccines was estimated. And the factors influencing their spread were identified. The information in this study can provide an important scientific basis for government decisions.

## Results

### Potential geographical distribution of illegal vaccines

**Figure 1:**
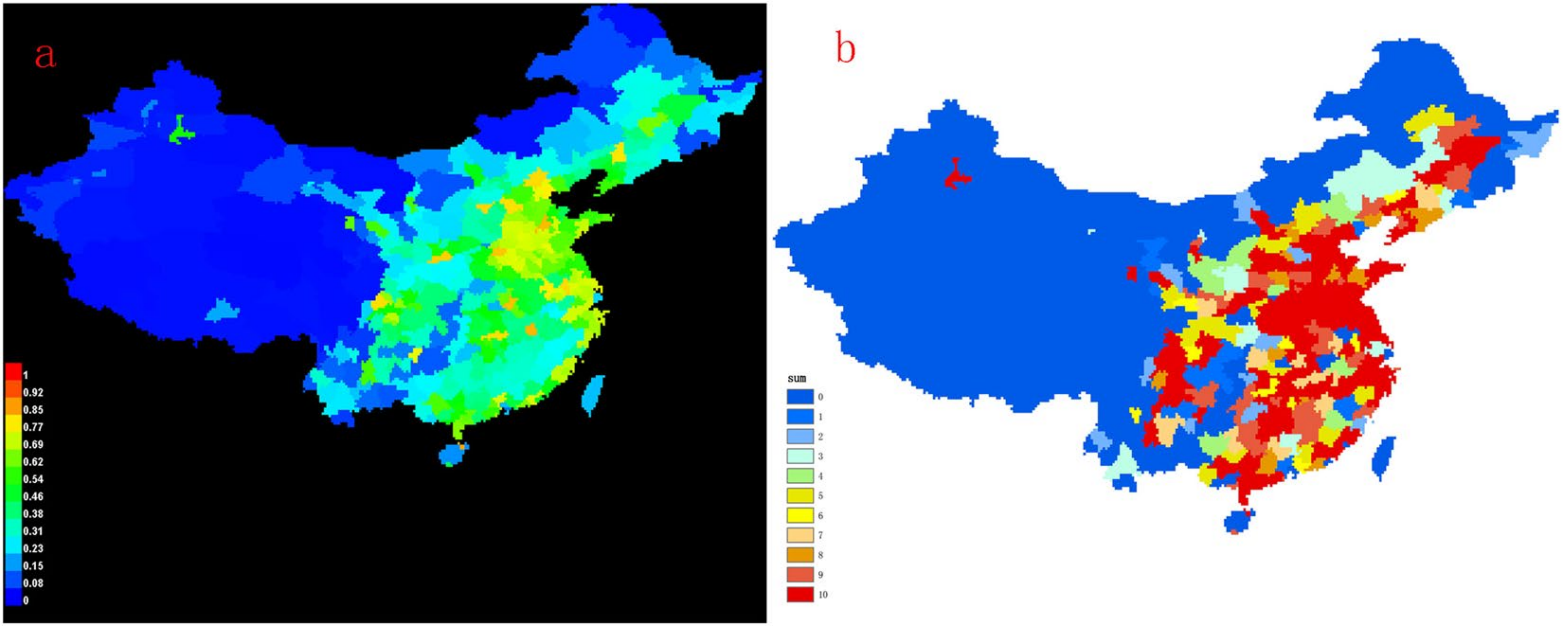
Suitability distribution map of illegal vaccines across China ((**a**) Maxent version 3.3.3k (http://www.cs.princeton.edu/) and (**b**) GARP version; ArcGIS also used.

The risk distribution map of illegal vaccines’ potential inflow regions in China, as shown in Fig. 1a, was obtained using MAXENT. The probability of illegal vaccines occurrence (P) of the areas potentially affected by illegal vaccines is between 0 and 1. The brighter the color is, the higher the distribution probability is ref. 26. The color blue means zero distribution. GARP was used to generate the binary map (Fig. 1b), which indicates the existence and absence of 0 and 1. The index (overlap) P refers to the number of projections for the existence of the model, used to indicate the degree of possibility of the existence of the forecast. As the two figures show, northern and eastern China was at a high risk of being affected by the illegal vaccines, while the probability of illegal vaccines occurrence was low in western China.

**Figure 2:**
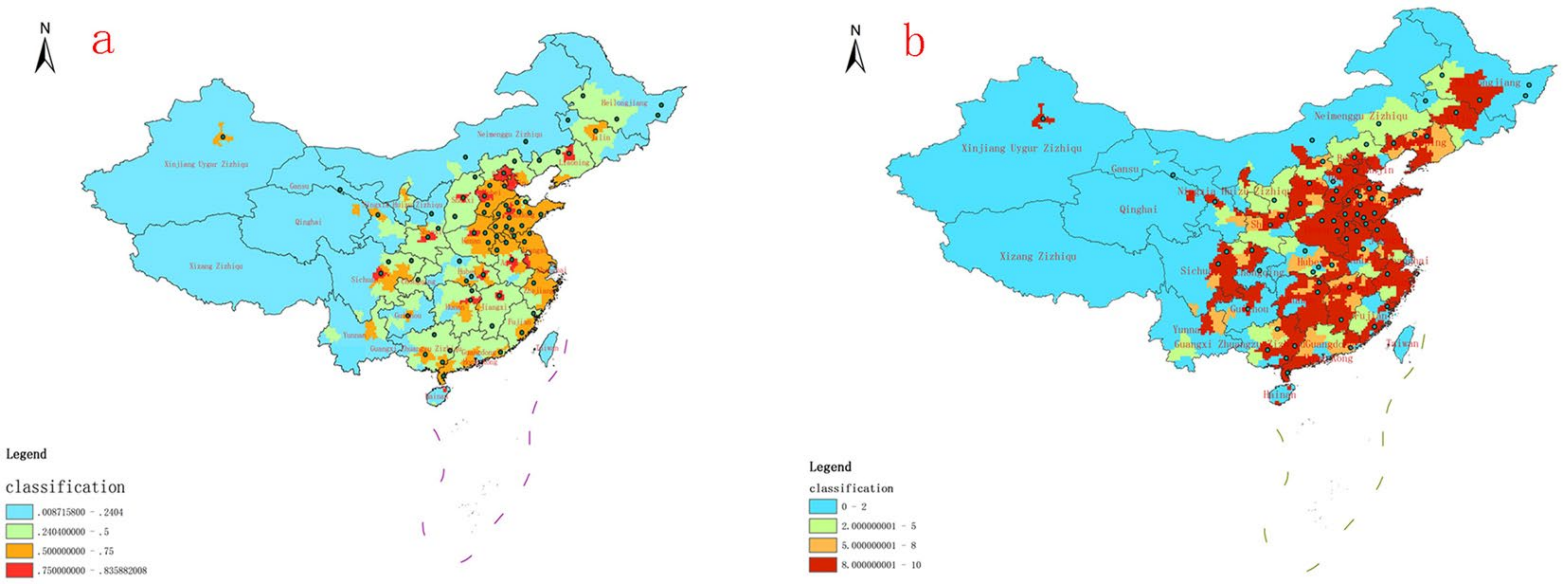
Spatial suitability distribution map of risk areas in China affected by vaccines involved. (**a**) The result of Maxent, (**b**) the result of GARP (Maps created in ArcGIS 10.2 (Environmental Systems Resource Institute, ArcMap Release 10.2, Environmental Systems Resource Institute, ArcMap Release 10.2, ESRI, Redlands, California)).

The suitability of the risk areas was evaluated using the Maxent model. The distribution map of suitable risk areas was then converted from ASCII to raster via ArcGIS’ format conversion tools, and appropriate thresholds were selected to classify spatial risk areas using the reclassification tool in Spatial Analyst Tools. A ‘10 percentile training presence logistic threshold’ was adopted in this study to define the minimum threshold of risk areas affected by illegal vaccines^27, 28^. The suitability level of the spatial distribution range of the risk areas for illegal vaccines in this paper was 0.2404. Therefore, the final set levels were highly suitable (P > 0.75), suitable (0.5 < P < 0.75), less suitable (0.2404 < P < 0.5) and not suitable (P < 0.2404), respectively. The spatial suitability distribution map of illegal vaccines’ risk areas in China was finally obtained (Fig. 2a). As with the Maxent (Maximum Entropy Model) results, a distribution map of suitable risk areas was also generated using the GARP (Genetic Algorithm for Rule set Production) model (Fig. 2b).

Both Maxent and GARP model have better forecasting accuracy on predicting potential risk areas of illegal vaccine. However, Maxent predicted the potential risk areas more accurately than other models such as GARP. Comparing the results of the two model predictions, the GARP model often creates a relatively larger and more continuous potential risk area, which partly over-predicts the fragmental habitats. The Maxent model adapted better to different levels of socioeconomic suitability and removed unreasonably fragmented distribution, better illustrating the potential distribution pattern of the illegal vaccine.

The results indicated that the vaccines involved had a wide potential geographic distribution in China. The highly suitable areas included South Central Hebei, North Shandong, Central Henan, Central Sichuan, Central Shanxi, Nanjing, North Jiangxi and Northeast Hunan. Those regions were mentioned in news reports, which indicates reflect a high predictive accuracy. Haikou was included in the predicted risk areas, but no reports indicated a problem there. The suitable risk areas included East and Central China, Inner Mongolia, part of North Urumqi in Xinjiang, East Gansu and some cities in Shaanxi; several cities in Qinghai, Yunnan and Ningxia, where there were no problems reported, were also included. Ningxia, Yunnan and Hainan were included in the less suitable areas, along with 24 provinces indicated in news reports; the potential risk to those three provinces may be unknown. The 72 points predicted in the map were the officially confirmed inflow points of the vaccines involved. As shown, the predicted areas covered nearly 80 counties and cities in 24 provinces such as Beijing, Henan and Shandong, which roughly conformed to the officially issued result. The models predicted the risk with 95.8% accuracy.

### Evaluation of models

**Figure 3:**
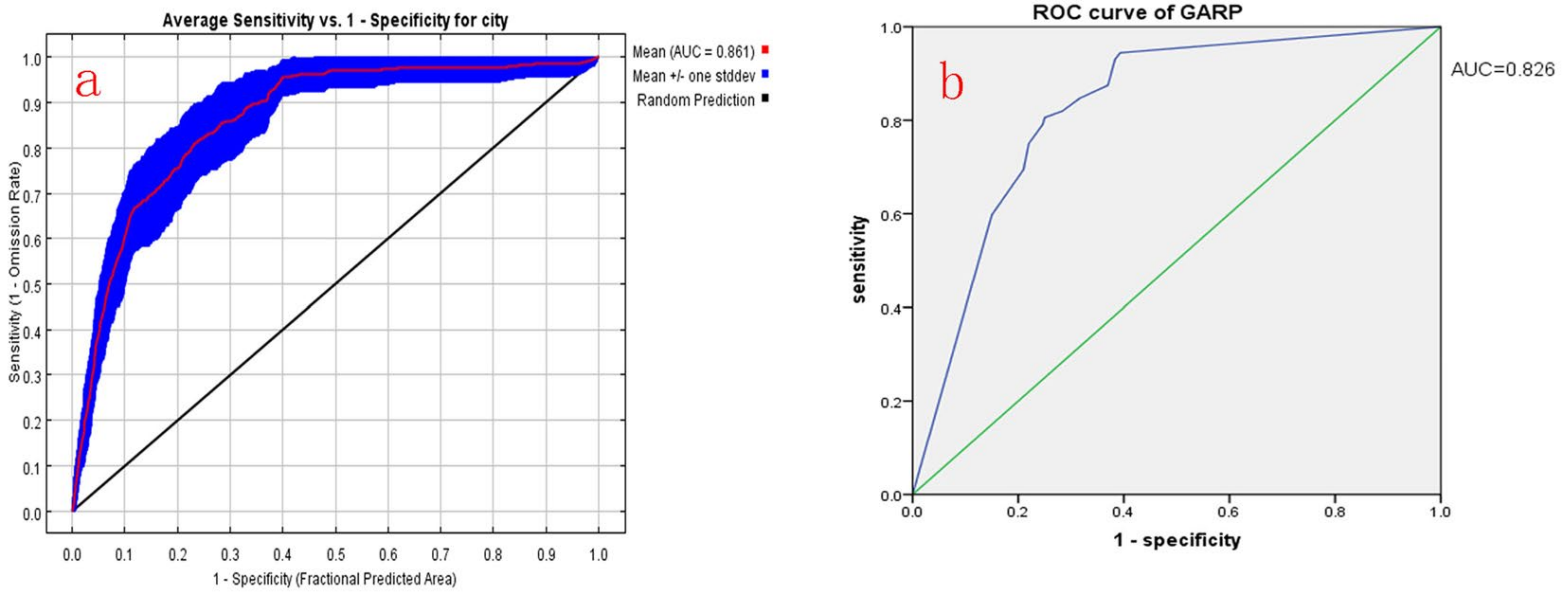
ROC curve of GARP model (**a**) and ROC curve of Maxent model (**b**).

We used the value of the area under the curve (AUC), which is under the Receiver Operating Characteristic curve (ROC), to assess the prediction performance of the GARP and Maxent models. High AUC values indicate a higher correlation between the entered socioeconomic variables and the geographic distribution of the species, a better ability to judge whether the species was distributed in a given area, and more accurate predictions. The model prediction result was evaluated using the ROC curve, and the area under the curve (AUC) was taken as a measure of the model’s predictive ability and was valued from 0 to 1. A higher AUC value indicates a less random species distribution (AUC = 0.5) and a greater correlation between environment variables and the model, i.e. a more accurate model^29^. The rough standard for evaluating model performance based on AUC value was as follows: When 0.9 < AUC < 1.0, the model’s prediction is excellent; when 0.8 < AUC < 0.9, the model’s prediction is good; when 0.7 < AUC < 0.8, the model’s prediction is ordinary; when 0.6 < AUC < 0.7, the model’s prediction is poor; and when 0.5 < AUC < 0.6, the model’s prediction is disqualified^30^. AUC is widely used to compare the performance of species distribution models^31–33^. GARP had a relatively lower AUC score (AUC = 0.826) (Fig. 3b), which indicates its lower ability in predicting potential distribution. Comparing with GARP, Maxent had a better performance with an AUC value of 0.861(Fig. 3a), and risk areas and non-risk areas in the region affected by illegal vaccines can be well distinguished.

### Socioeconomic variables’ contribution and variables’ response to suitability

**Figure 4:**
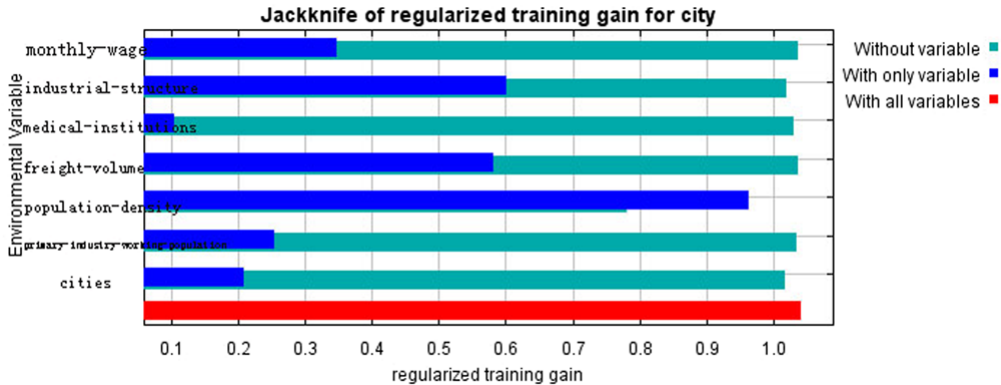
Maxent jackknife method (The jackknife figure reflected the impact of each variable on the entire model and reflected the function and signification of each variable in more detail. Light blue indicates the impact on the model if this variable is not included, and dark blue indicates the independent contribution of this variable to the model).

We considered each variable’s contribution to the model and each variable’s influence on the probability of illegal vaccines occurrence. The jackknife test was used to evaluate the relative importance of single explanatory variables included in the Maxent model. The environmental variable with the highest training gain when used in isolation was considered the highest relative influence on the model’s AUC^34^. Figure 4 shows the contribution of each variable to the model. As illustrated in the figure, the most influential factors were population density (people-density) and industrial structure data (industrial-structure), which included the percentage of employees in primary, secondary and tertiary industries. The contribution rate for all of these factors was greater than 0.6 (independent contribution of variable to model), indicating that population density and industrial structure data included more useful information than other variables. The contribution of provincial capitals (cities), freight tonnage (freight-volume) and medical institutions at and under the township level (medical-institutions) was moderate. When other variables, including average monthly wages (monthly-wage) (RMB) and primary industry personnel (primary-industry-working-population) were used independently, their contribution rates to the model were low, indicating that these variables did not contain much useful information.

**Figure 5:**
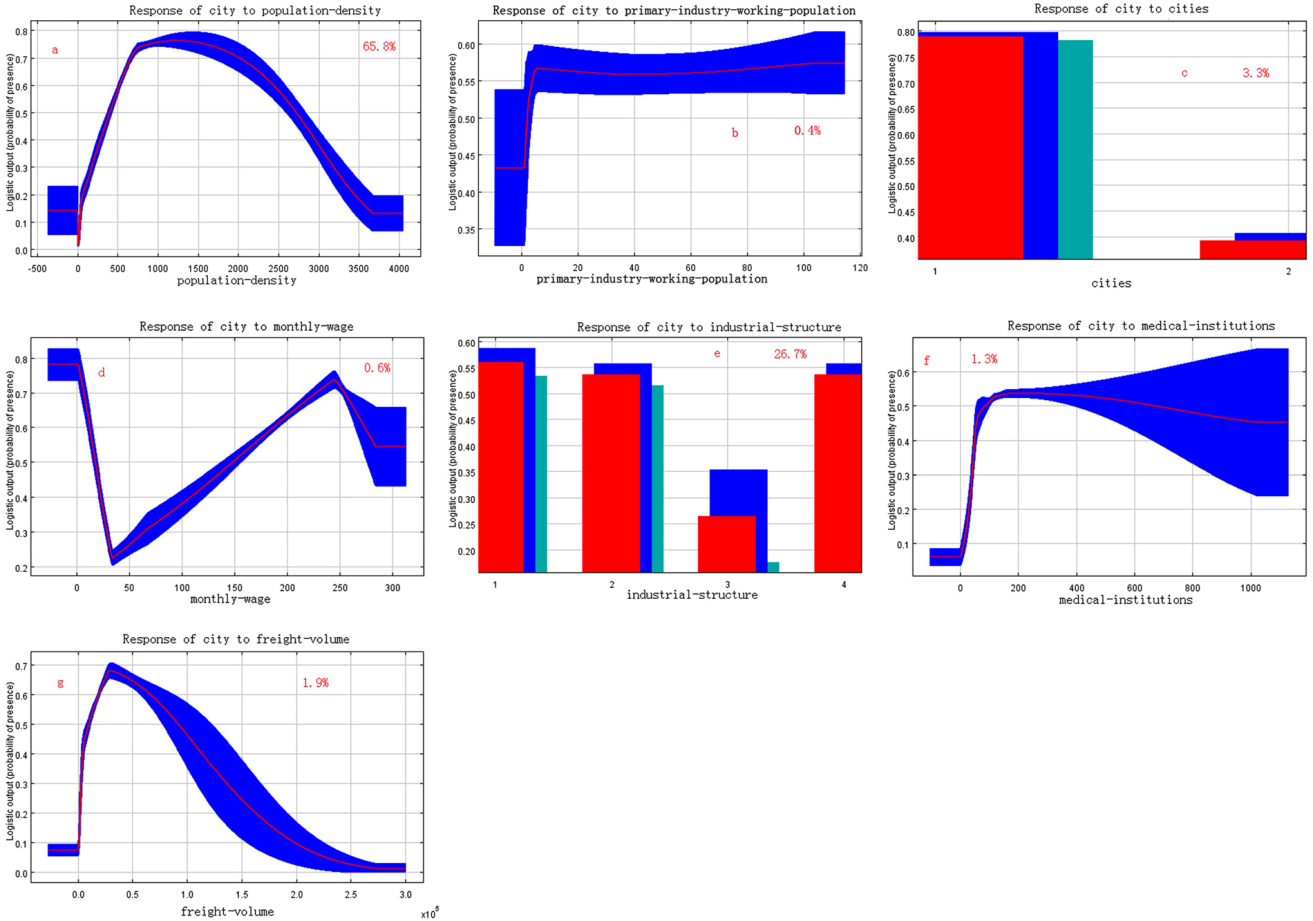
Response curves of Maxent jackknife method and environment variables ((**a**) people-density, (**b**) primary-industry-working-population, (**c**) cities, (**d**) monthly-wage, (**e**) industrial-structure, (**f**) medical-institutions, (**g**) freight-volume). The horizontal axis of the response curve indicates the value of the socioeconomic variable, and the vertical axis indicates probability that an area is at risk of being affected by illegal vaccines.

The response curve shows the quantitative relationship between the socioeconomic variables and the probability of illegal vaccines occurrence (suitability)^12^. The horizontal axis of the curve indicates the range of the environmental variable (rounded to a whole number), and the vertical axis indicates the logarithm of the environmental factor and distribution probability^26^. Figure 5 shows the response curve of each predictor variable. The response curve shows that the probability of illegal vaccines occurrence in a given area is higher until population-density (Fig. 5a) reaches 150 persons/sq.km. As population density increases, the probability decreases. When population density reaches 370 persons/sq.km, the probability remains steady; the risk increases to a peak value and then gradually decreases. If the primary-industry-working-population (Fig. 5b) exceeds 70,000 persons, the probability risk from the illegal vaccines was more than 0.51. The probability of illegal vaccines occurrence (Fig. 5c) was high, close to 0.72, and the probability of illegal vaccines occurrence outside the provincial capital cities was small, around 0.55. When monthly-wage exceeds RMB 400, the probability of risk gradually increases; when monthly-wage is RMB 2,400 to 2,700, the probability of risk from illegal vaccines decreases rapidly. For regions with a monthly-wage (Fig. 5d) above RMB 2,700, the probability of risk is 0.36. These results indicate that the impact scope suitable for illegal vaccines was broad and was usually concentrated in easily accessible areas with large populations, developed economies and high household consumption levels. Rural areas were also greatly impacted, as can be seen from the response curves of city to primary-industry-working-population, industrial-structure and medical-institutions. This is because rural areas have poor management and insufficient supervision. On the other hand, in most of western China, there was almost no risk because the area’s inaccessibility, underdeveloped economy and low level of medical care.

## Discussion

Since the vaccine incident, predicting the spatial distribution of problem vaccines’ potential risk areas, tracing the sale and use flows of those illegal vaccines and removing the remaining compromised vaccines from circulation, have all become hot-spot issues. In this case, the vaccines involved were the second type of vaccines, meaning that people were voluntarily inoculated and the expenses were paid by the patients or their guardians^35^. There are currently three^36^ management modes for this type of vaccines: (I) as with the first type of vaccines (required vaccines), a provincial-level health administration or disease control agency organizes the purchase and sale of vaccines, and the disease control agency controls the supply and manages the administration of the vaccines; (II) the city- or county-level health administration or disease control agency organizes the purchase and sale of vaccines; or (III) the township-level (community) vaccination units purchase the vaccines themselves. At the moment, there are also a number of problems with secondary vaccines. Supplies of such vaccines are chaotic. There are a variety of supply units for secondary vaccines. Many vaccination units purchase the vaccines themselves, and the purchasing channels for such vaccines are chaotic as well^37^. The incident in Jinan was caused by a chaotic supply system for secondary vaccines. Other contributing factors included problems with vaccine management at township medical institutions, including poor vaccine transportation and storage environments and poor grass-roots vaccine management. Furthermore, personnel had the opportunity to profit from small medical institutions’ insufficient capabilities and a poor understanding of vaccines. Since vaccine is a special type of drug, any problem at any point in the chain of manufacture, circulation and use could pose a threat to human life and health. Vaccine management directly affects vaccine quality and thus vaccination quality^38^. The illegal vaccine incident indicated that standardized vaccine management is critically important, and the lack of it must be taken seriously. It affects the physical and mental health of vaccinated groups, so a higher value must be placed on vaccine management to ensure vaccine quality and vaccination safety.

This study used the Maxent model and the GIS technique to estimate which areas in China could have been affected by the vaccine incident. The high-risk areas were defined, and the major socioeconomic factors affecting the spatial distribution of illegal vaccine risk areas were determined. The risk areas predicted by this study generally conformed to related news reports. The Maxent model was used in this study due to the following advantages: (1) known data can be entered only in species distribution data; (2) continuous variables and classified variables can be entered as environmental variables; (3) even in the circumstance of incomplete data, the sample size was smaller, and the difference between samples was great, the prediction accuracy of MAXENT was still stable and reliable; (4) Maxent can automatically generate a clear space habitat suitability map, a ROC curve and a missing error analysis curve for each factor; and (5) the importance of each environment variable can be evaluated using the jackknife method. (6) The modeling operation required two types of data: the actual geographic distribution point of target species, and the environmental variables of the study area. The Maxent model includes a self-checking function and can generate a ROC curve automatically. (7) The Maxent model can adapt to variations in environmental suitability and remove unreasonable fragmentation distribution and then visually illustrate the potential distribution pattern of illegal vaccines.

In this study, the area under curve (AUC) of the model prediction results reached 0.861, while the division of risk levels by region basically conformed to the current distribution status of areas at risk from compromised vaccines in China. Therefore, the results are credible. This paper estimated the areas of China likely to be affected by the vaccine incident, and Shandong, Hebei, Henan, Jiangsu and Anhui were identified as the major high-risk areas. Population density and industrial structure were the major socioeconomic factors affecting the spatial distribution of the risk areas. The regions which were most likely to be affected by illegal vaccines were accessible cities with high populations, developed economies and high household consumption levels. As shown in the response curves of city to primary-industry-working-population, industrial-structure and medical-institutions, rural areas were also very likely to be affected because of poor management and insufficient supervision. This study identified Ningxia, Yunnan and Hainan as high-risk areas, as well as correctly identifying the areas that were officially named as affected by the incident. These results may be due to the high number of rural areas in Ningxia and Yunnan. Rural medical institutions face problems in vaccine management, including poor vaccine transportation and poor grass-roots vaccine management. Furthermore, poor capabilities and a poor understanding of vaccines in rural facilities provide an opportunity for personnel to profit from these weaknesses. However, since Hainan has relatively developed regional economy, illegal vaccines may come from Guangdong.

Clearly, the management of secondary vaccines in China needs improvement. Strict governmental supervision should oversee the purchase of secondary vaccines, especially in rural areas. Furthermore, regulations regarding the cold-chain system of vaccine storage and transportation must be strictly adhered in order to ensure vaccine quality. Finally, the management of secondary vaccines needs to be monitored daily. Each vaccination unit should regularly report their purchase and cold-change storage of vaccines and vaccinations to the disease control agency. This would make it easier to supervise the use of vaccines and locate compromised vaccines more quickly^26^. The weaknesses of this research are as follows: (1) As the predicted results of different modelling methods may vary, the results need to be interpreted carefully in combination with the reality. In future research, multiple modeling methods could be used for comparison and integration. (2) This study was limited as a result of limited data, limited research time, and unsatisfactory data accuracy and integrity. (3) The data used for training the model may be a biased sample because of the simplex way to get data. The illegal vaccines were more likely to be reported in some areas, but not mentioned in the official information, which could lead to incorrect predictions. In future research, predictive ability could be perfected with data integrity.

## Materials and Methods

In order to use the Maxent (the freely downloadable Maxent Model version 3.3.3k (http://www.cs.princeton.edu/)) and GARP models to estimate the potential impact of illegal vaccines, two kinds of data were necessary: official vaccine inflow point data and data on environmental variables.

### Illegal vaccine inflow point data

**Figure 6:**
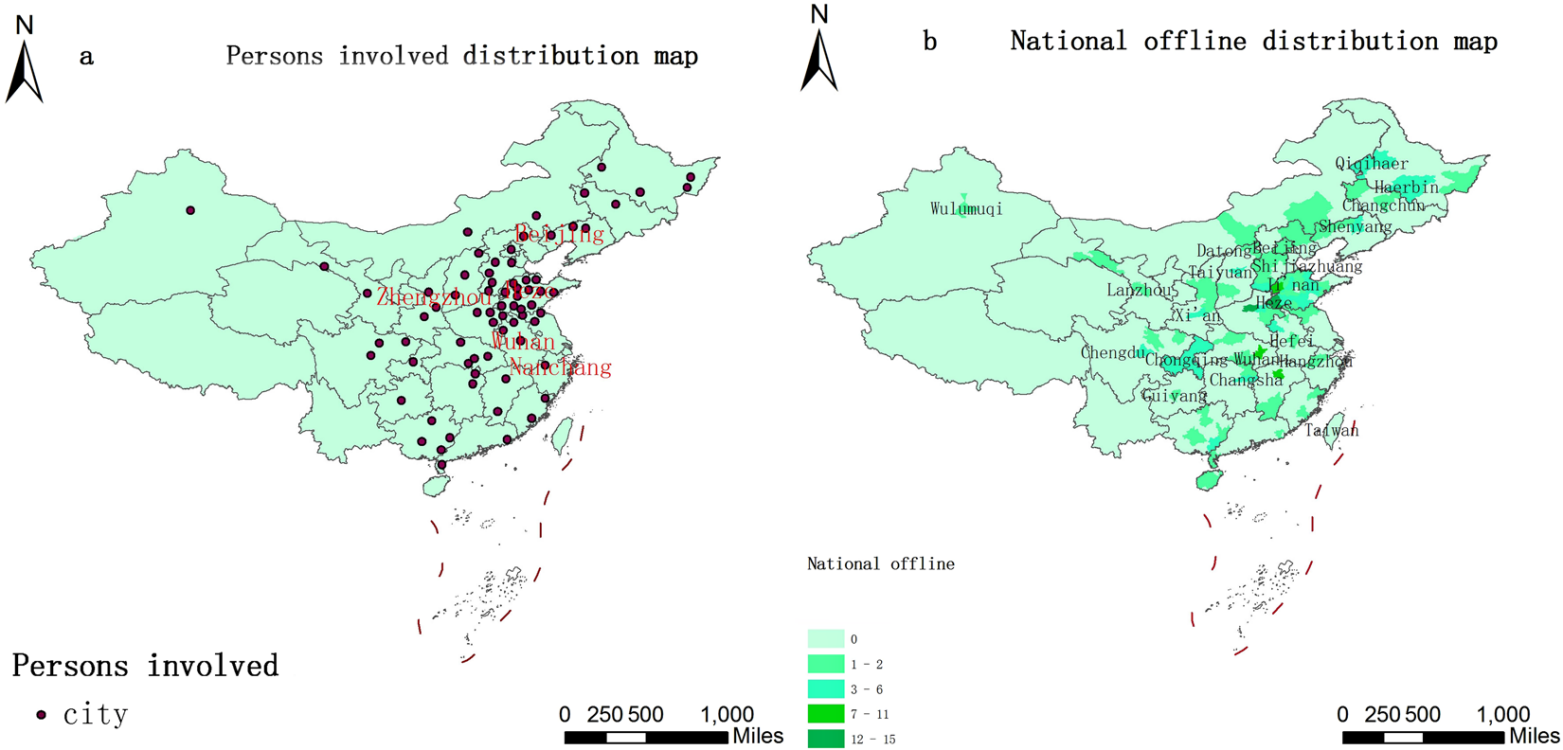
Persons involved distribution map (**a**) and National offline distribution map (**b**) (Maps created in ArcGIS 10.2 (Environmental Systems Resource Institute, ArcMap Release 10.2, Environmental Systems Resource Institute, ArcMap Release 10.2, ESRI, Redlands, California)).

This is the illegal vaccine inflow point data derived from official news reports^3^ (March 23) indicating the locations of the people involved. The vaccines involved flowed from Jinan, Shandong to the whole country. Many people were affected in the outflow cities; 15 people in Heze, Shandong, 13 in Zhengzhou, Henan, 11 in Wuhan, Hubei, 10 in Liaocheng, Shandong and 9 in Nanchang, Jiangxi. In all, people were affected in 72 different locations, including major cites impacted by illegal vaccines (Fig. 6).

### Socioeconomic variables

**Figure 7:**
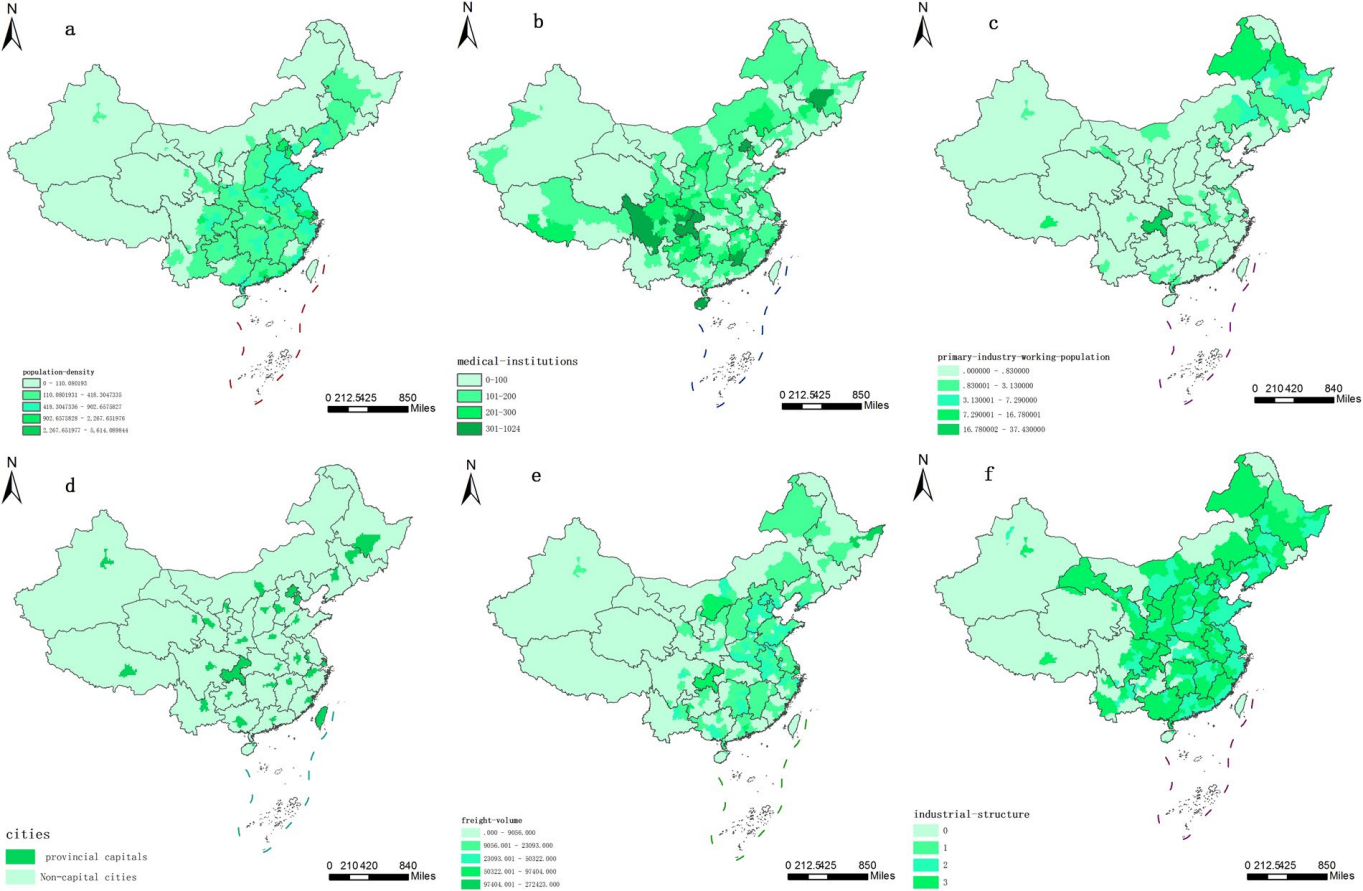
Socioeconomic variable data ((**a**) (population-density) National total population density map, (**b**) (medical-institutions) Distribution map of national medical institutions at and under the township level, (**c**) (primary-industry-working-population) primary industry personnel, (**d**) (cities) Distribution map of provincial capitals, (**e**) (freight-volume) Distribution map of freight tonnage, (**f**) (industrial-structure) Industrial structure data, including percentage of employees in primary, secondary and tertiary industries. Maps created in ArcGIS 10.2 (Environmental Systems Resource Institute, ArcMap Release 10.2, Environmental Systems Resource Institute, ArcMap Release 10.2, ESRI, Redlands, California)).

The selection of seven main socioeconomic variables influencing the probability of illegal vaccines occurrence was based on literatures review, correlation analysis and principal component analysis. According to the national population density map (Fig. 7a), illegal vaccines are more likely to circulate in areas with a higher population density, such as capital cities. Population density was therefore selected as a socioeconomic variable. The vaccines involved in this case were self-paid vaccines (optional or voluntary vaccines, which individuals can choose to receive at their own expense). According to the police report on the case^1^, some vaccination organizations had colluded with vaccine dealers and operations for a long time. The vaccination organizations sold the self-paid vaccines – which are easy to overstock and can even be sold at a discount close to the expiration date – and the law breakers sold these vaccines to regions and units with high demand, especially clinics and vaccination points in remote rural regions with poor government oversight. Therefore, the distribution conditions of primary industry personnel and medical institutions were also a factor impacting the circulation of illegal vaccines. Moreover, because the self-paid vaccines are not free to patients, residents’ income level may have influenced the circulation of the illegal vaccines. Therefore, average monthly wages in each city were also considered as a socioeconomic factor in this study.

According to news reports, socioeconomic variable data affecting the circulation of illegal vaccines can be classified into two types. The first type is continuous variable data, including population density (population-density) (person/sq.km.) (Fig. 7a), freight tonnage (freight-volume) (per 10,000 tons) (Fig. 7e), primary industry personnel (primary-industry-working-population) (per 10,000 people) (Fig. 2c), average monthly wages (monthly-wage) (RMB) and medical institutions at and under the township level (medical-institutions) (number) (Fig. 7a). The second category is classified variable data, including industrial structure data (industrial-structure) (Fig. 7f). This includes the percentage of employees in primary, secondary and tertiary industries and provincial capitals (cities) (Fig. 7d). These data were derived from the 2013 Chinese Statistical Yearbook. All the variables had to be discretized before being entered into the MAXENT model. The discretization results of the continuous socioeconomic variables are shown in Table 1.

**Table 1:** Division grade corresponding to each social variable.

Socioeconomic variable	Grade quantity	Intervals
National urban population density (population-density)	5	Person/sq.km.
Medical institutions at and under the township level throughout the country (medical-institutions)	4	Less than 100, 100–200, 200–300, and more than 300
Average monthly wages (monthly-wage)	4	Less than RMB 3,500, RMB 3,500–4,000, RMB 4,000–4,500 and more than RMB 4,500
Industrial structure	3	Percentage of employees in primary, secondary and tertiary industries
City data (cities)	2	provincial capital and non- provincial capital
Freight-volume	5	10,000 tons
Primary-industry-working-population	5	10,000 persons

All data layers had a unified WGS84 coordinate system and were converted into an ASCII file format as required by the Maxent model; a regular grid layer (326*250) with a pixel size of 0.19° covering all of mainland China was generated.

### Maxent

In 1957, Jaynes proposed the maximum entropy theory. This theory argues that an object with maximum entropy is closest to its real condition in known conditions, so the risk in making predictions is lower. In bio-ecology, this theory is applied as follows: in the absence of other constraints, one species will spread out until it is as close as possible to a uniform distribution^13, 33^. Therefore, when the survival conditions of a species are unknown, the most reasonable prediction for whether this species can survive in a specific location is 50% for existence and 50% for non-existence. This aroused concern in ecological circles^39^. Based on this principle, Phillips *et al*.^30^ used Java to compile software to predict the potential geographic distribution of a species. This software was used to accurately predict and evaluate species habitats^34^. The maximum entropy principle can also be used to speculate about unknown aspects of a target area from incomplete known information; for example, existing species distribution data and environmental data can be utilized to study the non-random relationship between environmental features of known species distribution areas and study areas. When certain limiting conditions (a set of constraints representing incomplete information about target areas—that is environmental variable)^33^ were met, maximum entropy can be used to predict the probable optimal distribution in a suitable habitat for a species^29^. The formula is expressed as follows:

1 $${\rm{P}}=\{p|{E}_{P}{f}_{j}={E}_{\tilde{P}}{f}_{j},j=\{1,\ldots ,k\}\}$$

2 $${p}^{\ast }={\rm{\arg }}\,\mathop{{\rm{\max }}}\limits_{p\in P}\,H(p)$$

If k features f_j_(j = 1, 2, …, k) exist in an ecological niche, the set of multiple constraint equations is called a constraint set, and the maximum entropy model p* is the model with maximum entropy in all models meeting the constraint set conditions. In this study, Maximum Entropy Species Distribution Modeling was obtained using the Maxent software.

Cross-validation was used to evaluate the accuracy of the predictive model. Seventy-five percent of the sample data was randomly selected for validation, and the remaining 25% of sample points were used to test the model’s predictive ability. In order to avoid random errors from affecting the selection of the validation and prediction samples, the Maxent model was run a total of 30 times, and the average value was used to calculate the potential geographic distribution. The file format was set for logistic output, which produces predicted probabilities of presence between 0 and 1, and it was scaled in a non-linear mode for explanation. The settings of the other parameters are shown in Table 2. The probability of illegal vaccines occurrence was affected by the sample design, such as sample size and observation time. The logic output for predicting the probability of illegal vaccines occurrence was based on this assumption: a typical area has a 0.5 probability of occurrence, and 0.5 is relatively random. If more information is known about an area, this value is adjustable. If p(x) is the raw output of environmental condition x, then the corresponding logistic value will be c p(x)/(1 + c p(x)), where c is a particular value (i.e. the index value of the entropy of raw distribution)^24^.

**Table 2:** Settings of partial parameters of Maxent model initialization.

Parameter name	Parameter value
Random test percentage	25%
Regularization multiplier	1.5
Replicates	30
Replicated run type	Subsample
Maximum iterations	5000
Adjust sample radius	3
Treads	5

### GARP

As a comparison, the GARP model was also used to estimate the potential distributions of illegal vaccines. The GARP model is a widely-used species distribution modeling tool. GARP (Genetic Algorithm for Rule set Production) prediction is based on the principle of genetic algorithms. First, Prediction data are randomly divided into training data (for training and building models) and testing data (for model quality testing), through the iterative rule set: selection, evaluation, validation, and acceptance or rejection. Four candidate sets of rules (atomic, logistic regression, bioclimatic envelope and negated bioclimatic envelope) are used to develop a method, which is then applied to the training data. This process generates a rule for determining whether the accuracy of the prediction can be improved based on the validation data. This algorithm usually runs several times or terminates according to the convergence condition, and finally forms a model composed of different rules, which can predict the potential distribution of the species^40^.
